# The association between socioeconomic factors and the success of decolonization treatment among individuals diagnosed with methicillin-resistant *Staphylococcus aureus*: A cohort study from 2007 to 2020

**DOI:** 10.1017/ice.2023.32

**Published:** 2023-10

**Authors:** Mette Assenholm Kristensen, Julia Skov Abrantes, Hanne Irene Jensen, Christian Backer Mogensen, Jens Søndergaard, Jens Kjølseth Møller

**Affiliations:** 1 Department of Clinical Microbiology, Lillebaelt Hospital, University Hospital of Southern Denmark, Vejle, Denmark; 2 Department of Regional Health Research, University of Southern Denmark, Odense, Denmark; 3 Department of Quality, Lillebaelt Hospital, University Hospital of Southern Denmark, Vejle, Denmark; 4 Department of Anesthesiology and Intensive Care, Lillebaelt Hospital, University Hospital of Southern Denmark, Vejle, Denmark; 5 Department of Emergency Medicine, Hospital Sønderjylland, University Hospital of Southern Denmark, Aabenraa, Denmark; 6 Research Unit of General Practice, Department of Public Health, University of Southern Denmark,Odense, Denmark

## Abstract

**Objectives:**

To examine associations between socioeconomic factors and (1) adherence to methicillin-resistant *Staphylococcus aureus* (MRSA) posttreatment follow-up swab sampling after 1 and 6 months and (2) successful decolonization treatment.

**Design:**

Cohort study with 2 years of follow-up. Data on patients diagnosed with MRSA were extracted from a regional MRSA database and national registries. We used a cluster-based logistic regression model to estimate the adjusted odds ratios (aOR) and 95% confidence interval (CI) for associations between socioeconomic factors and decolonization treatment.

**Setting:**

Danish primary health care.

**Results:**

The rate of adherence to posttreatment follow-up swab sampling among 2,536 cases 1 month after decolonization treatment was 66% (95% CI, 64%–68%), and it decreased to 30% (95% CI, 28%–32%) after 6 months. Living in intermediate municipalities (76–159 inhabitants/km2) or having retired were associated with completed posttreatment follow-up swabs 1 month after decolonization treatment: aOR, 1.40 (95% CI, 1.2–1.74) and aOR, 2.67 (95% CI, 1.16–6.13), respectively. The rate of successful decolonization treatment 2 years after initiating treatment was 36% (95% CI, 34%–38%). Factors associated with successful decolonization treatment included individuals with higher education (aOR, 1.62; 95% CI, 1.22–2.15), early retirees (aOR, 1.63; 95% CI, 1.12–2.38), those living in intermediate municipalities (ie, 160–900+ inhabitants/km2; aOR, 1.35; 95% CI, 1.08–1.68), and those living in predominantly urban municipalities (ie, 160–900+ inhabitants/km2; aOR, 2.04; 95% CI, 1.5–2.76).

**Conclusions:**

Disparities in the effect of decolonization treatment and adherence to MRSA follow-up sampling among MRSA-positive individuals appear to be largely explained by the level of education, area of residence, and employment status.

Antimicrobial drug resistance poses a major global threat to human health. Methicillin-resistant *Staphylococcus aureus* (MRSA) is a leading cause of death associated with antimicrobial resistance.^1^ MRSA carriage also has considerable social and psychological consequences for those diagnosed with it.^2^ Despite better overall health, health inequalities still exist in European countries.^3^ Studies have reported higher rates of communicable diseases among people with lower socioeconomic status.^4,5^ In some high-income countries, antibiotic-resistant infections are likelier among the unemployed and low-income populations living in deprived neighborhoods.^4,6^ Therefore, social factors related to infectious diseases have also become a public health priority in high-income countries to limit the disease burden on individuals and to decrease the spread of infectious diseases in communities and healthcare systems.^7^ Overcrowding, low education, an urban living environment, and recent immigration seem to increase MRSA rates.^8,9^ Length of education is strongly linked to health literacy skills and, consequently, to the ability to act upon health information. Furthermore, low health literacy is strongly associated with poor adherence to treatment.^10,11^

Denmark has small-scale social and economic inequalities. The public healthcare system is mainly tax funded and free for all citizens.^12^ To maintain a low prevalence of disease caused by MRSA in Denmark, national guidance on preventing the spread of MRSA includes offering sampling for MRSA and decolonization treatment for MRSA-positive individuals.^13^ All household members of MRSA carriers are also offered decolonization treatment.^13^ Risk factors for failed MRSA carriage decolonization treatment are typically based on individual risk factors (eg, skin lesions and throat carriage).^14^ However, little is known about the importance of socioeconomic factors for adherence to follow-up swab sampling and MRSA cure rates after decolonization treatment. Therefore, we examined whether socioeconomic factors are associated (1) with adherence to MRSA posttreatment follow-up swab sampling after 1 and 6 months and (2) with successful decolonization treatment. We hypothesized that low socioeconomic status is associated with lower rates of adherence to MRSA posttreatment follow-up swab sampling and a lower success rate of decolonization treatment of MRSA carriage.

## Methods

### Study design and setting

We conducted a cohort study using prospectively collected register-based information at the individual level for MRSA-diagnosed individuals (asymptomatically colonized or infected with MRSA) in the Region of Southern Denmark (RSD) (population, ∼1.2 million). The follow-up time was 2 years. Based on the first Danish national guidance on preventing the spread of MRSA from 2006 and a national reorganization (Danish Municipal Reform) of all municipalities and regions in 2007, we chose the study period 2007–2020. In the RSD, decolonization treatment and follow-up swab sampling are mainly handled by general practice staff, with guidance from the department of clinical microbiology upon request. MRSA carrier treatment is free for all household members. Public interpretation services are available for foreign-speaking citizens. The main part of the study period was conducted in an endemic setting with a low prevalence of MRSA (ie, 49.5 asymptomatic colonization or clinically apparent infections with MRSA per 100,000 inhabitants).^15^ However, 2 nosocomial outbreaks have been described.^16,17^ The Danish national guidance on preventing the spread of MRSA was revised in 2012 (ie, primary changes: livestock MRSA added, routine follow-up swab sampling should be taken at 6 months instead of 12 months after decolonization treatment) and in 2016 (minor revision). In Denmark, all citizens with a permanent residence are assigned a 10-digit Central Personal Register number, which is used in all Danish public registries, allowing the linkage of data on individuals. Furthermore, families are assigned a unique family identifier.^18^

This study has been reported according to Strengthening the Reporting of Observational Studies in Epidemiology (STROBE) for antimicrobial stewardship.^18^ Permission to store data was registered in Record of Data Processing Activities in the RSD (no. 20/25135), and the study was approved by the Danish Patient Safety Authority (no. S-31-1521-375). It was not necessary to obtain ethical approval for this project (no. S-20192000-155).

### Participants

We included MRSA-diagnosed patients who were treated for 5 or 10 days (throat carriers) with 2% mupirocin nasal ointment (Bactroban nasal) in the primary healthcare sector from 2007 to 2018. Patients were included from the day the prescription of the initial decolonization treatment was redeemed. Information on cases with later colonization or infection with MRSA (relapse) after successful decolonization treatment was not included in the analysis. We excluded individuals who died, moved from the region, or left Denmark within 2 years of follow-up. In the analysis of success rate, we excluded participants who did not have posttreatment follow-up swab samples taken after 6 months. MRSA-diagnosed individuals were identified using a regional MRSA database. Statistics Denmark replaced Central Personal Register numbers with unique identifier numbers (pseudo-anonymized), and the study population (MRSA-diagnosed individuals) was matched with national registry data.

### Decolonization

In Denmark, MRSA carriage is treated with a topical application of 2% mupirocin to the anterior nose twice a day, combined with daily bathing with 4% chlorhexidine gluconate and environmental cleaning for 5 days. In cases of throat carriage, the treatment period is extended to 10 days and retreatment is recommended in case of treatment failure. Treatment is not recommended for children aged <2 years or for individuals with day-to-day contact with live pigs. There is no general recommendation to test household members before beginning treatment. Posttreatment MRSA follow-up swab samples are recommended 1 and 6 months after treatment. MRSA carriage treatment is considered effective when swab samples taken from the nose and throat are negative 6 months (or later) after decolonization treatment.^13^

### Outcome and exposure variables

**Fig. 1.**
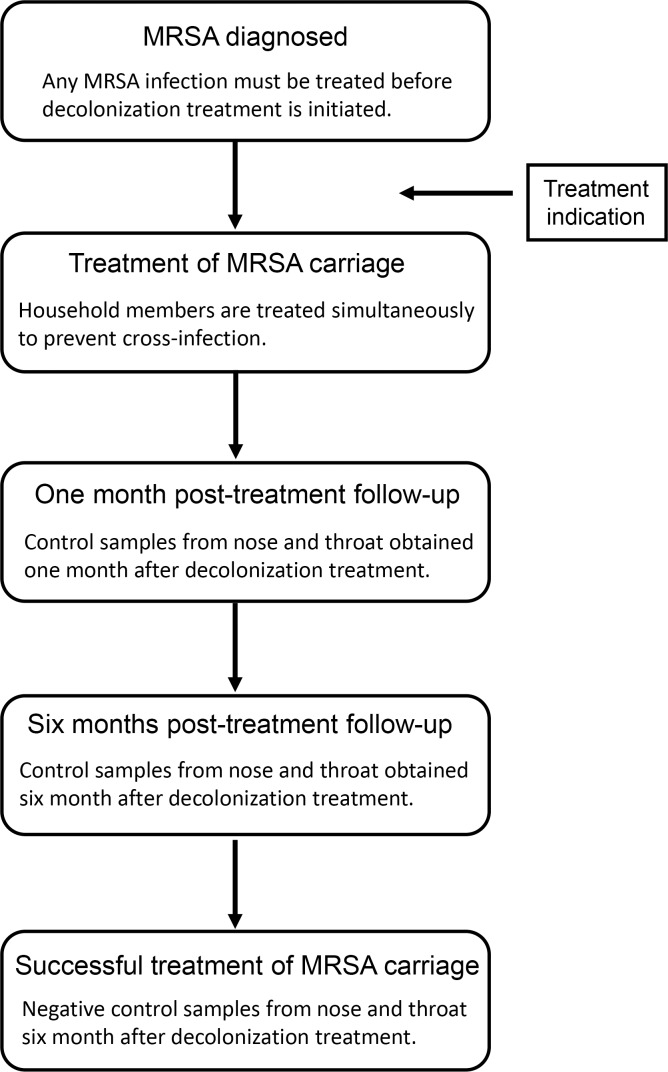
Treatment of MRSA carriage: Posttreatment follow-up and successful decolonization treatment.

For each patient with laboratory-confirmed MRSA, we collected information on sampling dates and laboratory results of all samples examined for MRSA in the regional MRSA database, which includes samples taken within hospitals and the community. Laboratory examinations of MRSA were done using either rapid screening of samples with polymerase chain reaction **(**PCR) and/or routine culture methods for detection of MRSA in clinical samples according to the European quality standard.^16,17^ MRSA posttreatment follow-up swab sampling was defined as sets of MRSA cultures from the nose and throat 1 and 6 months after decolonization treatment. Successful decolonization treatment was defined as a set of negative MRSA samples from the nose and throat at least 6 months after completing the decolonization treatment and before the end of the 2-year follow-up. Figure 1 briefly describes the process and definitions for treatment and posttreatment follow-up swab sampling and successful decolonization treatment. We obtained the prescription redemption dates for 2% mupirocin nasal ointment and possible systemic antibiotic treatment with a combination of 2 antibiotics given perorally for MRSA carriage in Denmark (eg, fusidic acid, rifampicin, and clindamycin) from the National Prescription Registry.

For each MRSA-diagnosed patient, we retrieved information on exposure variables on socioeconomic status from 4 validated national registries available in Statistics Denmark and reports published by Local Government Denmark (an interest organization of the 98 Danish municipalities) describing inhabitants per square kilometer for each municipality.^19–24^ The definitions of each explanatory variable examined are listed in Table 1. We considered these socioeconomic measures to cover important factors, in agreement with earlier studies.^4,6,8,25^ Categories for household crowding were based on former similar studies.^26^ The cutoff for groupings of family income (tertiles), education, and occupation was based on recommended categories from Statistics Denmark and the Odense Patient Data Explorative Network. The population density was classified into 3 categories: 0–75 inhabitants/km^2^ (predominantly rural municipality), 76–159 inhabitants/km^2^ (intermediate municipality), and 160–900+ inhabitants/km^2^ (predominantly urban municipality).

**Table 1.** Variables Describing Socioeconomic Factors

Variable	Definition	Data Administrator and Data Source
Disposable family income	The amount of money that the household has available for spending and saving after income taxes and interest expenses has been accounted for.	Statistics Denmark: The Income Statistics Register
Highest education completed	The highest education that a person has completed at any given point in time.	Statistics Denmark: The Population Education Register
Employment status	Attachment to the labor market from age 15 years. Includes all persons having an income during the year chargeable to income tax in Denmark.	Statistics Denmark: The Register-based Labour Force Statistics
Length of residence in Denmark	Years since immigration to Denmark or birthdate if born in Denmark	The Danish Civil Registration System
Household crowding	Residential size (m^2^) and the number of individuals living at the same address (family identifier).	Statistics Denmark: The Central Register of Buildings and Dwellings and The Danish Civil Registration System
Population density	Inhabitants per square kilometer	Statistics Denmark: The Danish Civil Registration System and an analyst note published by Local Government Denmark describing inhabitants per square kilometer for each municipality in Denmark

### Confounder variables and participant characteristics

Confounders were variables of importance for successful decolonization treatment in earlier studies.^14^ The number of decolonization treatments and systemic antibiotics was measured during an individual’s 2-year follow-up. Information about foreign bodies and somatic disorders was collected 5 years retroactively from study entry. The remaining variables were measured in the same year in which the first decolonization treatment was given. Data on somatic and mental disorders (ICD codes) and foreign bodies (treatment codes) were obtained from the National Patient Registry covering all admissions and outpatient visits to Danish hospitals.^27,28^ Data on systemic antibiotics and repeated decolonization treatment were obtained from the National Prescription Registry.^29^ The variable “MRSA-diagnosed in the household” was created using information on household relations (unique family identifier) and MRSA-diagnosed individuals identified from the regional MRSA database. Data on throat carriage were also obtained from the MRSA database.

### Statistical analysis

We descriptively estimated the cumulative incidence proportion of adherence to follow-up swab samples taken 1 and 6 months after decolonization treatment and successful decolonization treatment. All proportions were calculated with a 95% confidence interval (CI). Using the unadjusted odds ratio (OR) with 95% CI, we assessed the association between socioeconomic factors (ie, household crowding, education, family income, occupation, length of residence in Denmark, and population density) and (1) adherence to MRSA posttreatment follow-up swab sampling 1 and 6 months after decolonization treatment and (2) successful decolonization treatment, univariate analyses. To estimate the adjusted OR, we used a cluster-based logistic regression model. In this multivariable model, we adjusted for age, sex, MRSA diagnosed in the household, throat carriage, and number of decolonization treatments. Due to the low occurrence of somatic disorders, mental disorders, use of foreign bodies, and systemic antibiotics, these variables were not included in the adjusted analysis. *P* values < .05 (2-sided) were deemed statistically significant. Individuals lost to follow-up were excluded from the analysis. Statistical analyses were conducted using Stata version 17 software (StataCorp, College Station, TX).

## Results

**Fig. 2.**
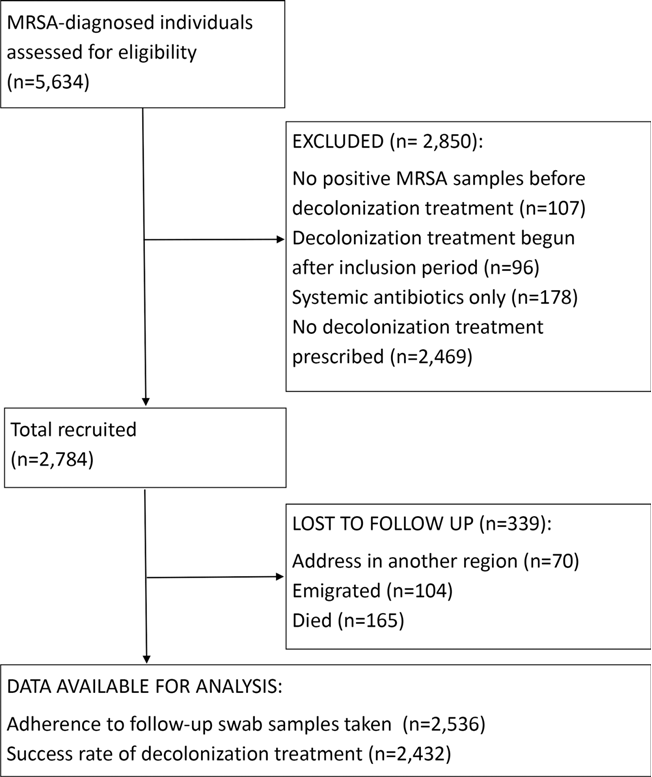
Participants flow diagram.

We extracted 5,634 MRSA-diagnosed individuals from the regional MRSA database. The main cause of exclusion was no treatment (n = 2,743). When comparing baseline socioeconomic characteristics of treated and untreated individuals, decolonization treatment was less likely to be initiated when individuals were aged 0–35 years, were recent immigrants, were male, and were employed (Supplementary Files 1 and 2 online). We included 2,536 individuals in the analysis of adherence and 2,432 individuals in the analysis of the success rate of MRSA clearance (Fig. 2). Table 2 shows the baseline demographic and clinical characteristics. The largest group of individuals was aged <34 years. Sex was almost equally distributed. Overall, 1,548 (61%) of MRSA-positive participants had only 1 course of treatment. Somatic disorders, foreign bodies, and mental disorders ranged from 1% to 2.3%. Approximately half of the study population had 1 or more culture-positive household contact.

**Table 2.** Baseline Demographic and Clinical Characteristics for Individuals Treated for MRSA Carriage^a^

Demographic and Clinical Characteristics	Total (N = 2,536) No. (%)
**Age group**	
0–34 y	1,311 (51.7)
35–49 y	449 (17.7)
50–64 y	423 (16.7)
65–79 y	266 (10.5)
≥80 y	72 (2.8)
**Sex**	
Male	1,210 (48)
Female	1,311 (52)
Throat carriage	1,394 (55)
**MRSA diagnosed in a household**	
1 individual	1,426 (56.2)
2 individuals	618 (24.4)
3 individuals	292 (11.5)
≥4 individuals	185 (7.3)
Unknown carriage household	15 (0.6)
**Decolonization treatment**	
1 treatment	1,548 (61.0)
2 treatments	640 (25.2)
3 treatments	227 (9)
≥4 treatments	121 (4.8)
Systemic antibiotic decolonization treatment^b^	294 (11.6)
**Somatic disorder**	
Diabetes mellitus	26 (1.0)
Chronic obstructive pulmonary disease	50 (2)
Chronic skin disease	33 (1.3)
Chronic wounds	25 (1)
Renal disease	19 (0.8)
Mental disorder	57 (2.3)
Foreign bodies	55 (2.2)

Note. MRSA, methicillin-resistant *Staphylococcus aureus.*

a Decolonization treatment: mupirocin nasal ointment 2% and body wash using chlorhexidine soap 4% for 5 or 10 days (throat carriage).

b Rifampicin, fusidic acid, clindamycin.

The rate of adherence to national guidelines advising MRSA follow-up swab sampling taken 1 month after treatment was 66% (95% CI, 64%–68%), and it decreased to 30% (95% CI, 28%–32%) after 6 months. Examining the relationship between socioeconomic factors and adherence to MRSA posttreatment follow-up swab sampling 1 month after decolonization treatment, we found that living in intermediate municipalities and being retired were associated with significantly higher rates of adherence (Table 3a) compared to predominantly rural municipalities. A significantly higher degree of adherence to MRSA follow-up swab sampling 6 months after decolonization treatment was only associated with living in areas with >160 citizens/km^2^ (Table 3b).

**Table 3a.** Cluster Analysis in a Multivariable Logistic Regression Model Analyzing the Association Between Socioeconomic Factors and Adherence to MRSA Posttreatment Follow-Up Swab Sampling After 1 Month^a^

Variable	Incidence Proportion (N = 2,536)	Crude Analysis 1 Month		Adjusted Analysis^b^ 1 Month	
	No. (%)	OR (95% CI)	*P* Value	aOR (95% CI)	*P* Value
**Employment status**					
Employed	1,160 (62)	1 (reference)		1 (reference)	
Student	183 (67)	1.28 (0.92–1.78)	.15	1.28 (0.85–1.91)	.23
Unemployed or welfare payment	225 (70)	1.44 (1.06–1.96)	.02	1.27 (0.90–1.77)	.17
Early retirement^c^	174 (67)	1.28 (0.91–1.79)	.16	1 (0.67–1.49)	.98
Retirement	310 (75)	1.92 (1.44–2.55)	<.001	2.67 (1.16–6.13)	.02
Other^d^	484 (70)	1.43 (1.14–1.79)	.002	1.16 (0.87–1.53)	.31
Data missing	0 (0.0)				
**Household income**					
Low tertile	841 (64)	1 (reference)		1 (reference)	
Middle tertile	840 (67)	1.14 (0.93–1.39)	.21	1.10 (0.86–1.41)	.46
High tertile	840 (69)	1.22 (0.99–1.49)	.06	1.24 (0.96–2.59)	.10
Data missing	15 (0.6)				
**Student**					
Lower secondary school	656 (67)	1 (reference)		1 (reference)	
Upper secondary school	863 (63)	0.87 (0.70–1.07)	.19	0.95 (0.74–1.21)	.66
Postsecondary school	378 (69)	1.13 (0.86–1.49)	.37	1.15 (0.84–1.56)	.39
Unknown	639 (68)	1.08 (0.86–1.37)	.50	1.19 (0.91–1.56)	.20
Data missing	0 (0.0)				
**Household crowding**					
≤20 m^2^ per person	2,179 (67)	1 (reference)		1 (reference)	
>20 m^2^ per person	239 (70)	0.85 (0.64–1.14)	.28	1.06 (0.71–1.6)	.77
Data missing	118 (4.7)				
**Population density**					
0–75 inhabitants/km^2^	840 (63)	1 (reference)		1 (reference)	
76–159 inhabitants/km^2^	1,283 (70)	1.37 (1.14–1.64)	.001	1.40 (1.12–1.74)	.003
160–660 citizens per inhabitants/km^2^	398 (64)	1.07 (0.83–1.37)	.62	1.06 (0.79–1.42)	.69
Data missing	15 (0.6)				
**Length of residence in Denmark**					
≤5 y	533 (67)	1 (reference)		1 (reference)	
>5 y	1,988 (67)	0.99 (0.81–1.21)	.99	0.94 (0.31–1.23)	.68
Data missing	15 (0.6)				

Note. MRSA, methicillin-resistant *Staphylococcus aureus*; OR, odds ratio; CI, confidence interval; aOR, adjusted OR.

a Follow-up swab samples from nose and throat (same date) 1–5 months after decolonization treatment.

b Adjusted for age, sex, number of decolonization treatments, MRSA-diagnosed in the household, and throat carriage.

c Voluntary early retirement of individuals partly depending on self-financing and age >60 years.

d Other includes individuals who have no connection to the labor market or with little connection to the labor market. Individuals in the other group do not receive welfare payments or education. Children aged <5 years are included in this group.

**Table 3b.** Cluster Analysis in a Multivariable Logistic Regression Model Analyzing the Association Between Socioeconomic Factors and Adherence to MRSA Posttreatment Follow-Up Swab Sampling After 6 Months^a^

Variable	Incidence Proportion (N = 2,536)	Crude Analysis 6 Months		Adjusted Analysis^b^ 6 Months	
	No. (%)	OR (95% CI)	*P* Value	aOR (95% CI)	*P* Value
**Employment status**					
Employed	1,160 (25)	1 (reference)		1 (reference)	
Student	183 (32)	1.37 (0.97–1.92)	.07	1.26 (0.86–1.87)	.24
Unemployed or welfare payment	225 (30)	1.28 (0.93–1.75)	.13	1.16 (0.82–1.65)	.41
Early retirement^c^	174 (39)	1.89 (1.36–2.63)	<.001	1.34 (0.90–1.99)	.15
Retirement	310 (42)	2.10 (1.62–2.73)	<.001	1.26 (0.59–2.71)	.55
Other^d^	484 (0.28)	1.15 (1.62–2.72)	.25	1.06 (0.79–1.41)	.71
Data missing	0 (0.0)				
**Household income**					
Low tertile	841 (31)	1 (reference)		1 (reference)	
Middle tertile	840 (28)	0.85 (0.69–1.05)	.14	0.90 (0.69–1.17)	.42
High tertile	840 (30)	0.94 (0.76–1.15)	.54	1.01 (0.78–1.32)	.93
Data missing	15 (0.6)				
**Education**					
Lower secondary school	656 (31)	1 (reference)		1 (reference)	
Upper secondary school	863 (28)	0.89 (0.71–1.11)	.29	1.02 (0.80–1.31)	.84
Postsecondary school	378 (35)	1.20 (0.92–1.58)	.18	1.33 (0.98–1.79)	.06
Unknown	639 (28)	0.87 (0.69–1.11)	.27	1.11 (0.84–1.47)	.45
Data missing	0 (0.0)				
**Household crowding**					
≤20 m^2^ per person	2,179 (30)	1 (reference)		1 (reference)	
>20 m^2^ per person	239 (34)	0.82 (0.62–1.08)	.16	0.80 (0.51–1.23)	.31
Data missing	118 (4.7)				
**Population density**					
0–75 inhabitants/km^2^	840	1 (reference)		1 (reference)	
76–159 inhabitants/km^2^	1,283	1.28 (1.05–1.56)	.01	1.22 (0.96–1.54)	.10
160–900+ inhabitants/km^2^	398	1.92 (1.49–2.48)	<.001	1.79 (1.32–2.44)	<.001
Data missing	15 (0.6)				
**Length of residence in Denmark**					
≤5 y	533 (26)	1 (reference)		1 (reference)	
>5 y	1,988 (31)	1.31 (1.06–1.63)	.01	1.80 (0.82–1.43)	.59
Data missing	15 (0.6)				

Note. MRSA, methicillin-resistant *Staphylococcus aureus*; OR, odds ratio; CI, confidence interval; aOR, adjusted OR.

a Follow-up swabs from nose and throat (same date) 6–12 months after decolonization treatment.

b Adjusted for age, sex, numbers of decolonization treatments, MRSA-diagnosed in the household, and throat carriage.

c Voluntary early retirement of individuals partly depending on self-financing and an aged >60 y.

d Other includes individuals who have no connection to the labor market or with little connection to the labor market. Individuals in the other group do not receive welfare payments or education. Furthermore, children aged <5 years are included in this group.

The cumulative incidence proportion of successful decolonization treatment was 36% (95% CI, 34%–38%). Postsecondary school (short-cycle tertiary education or university degree completed), living in predominantly urban and intermediate municipalities, and individuals who retired early (voluntary early retirement of individuals partly depending on self-financing and age >60 years) were associated with a significantly higher rate of successful decolonization treatment (Table 4).

**Table 4.** Cluster Analysis in a Multivariable Logistic Regression Model Analyzes the Association Between Socioeconomic Factors and Successful Decolonization Treatment^a^

Variable	Incidence Proportion (N = 2,432)	Crude Analysis		Adjusted Analysis^b^	
	No. (%)	OR (95% CI)	*P* Value	aOR (95% CI)	*P* Value
**Employment status**					
Employed	1,103 (31)	1 (reference)		1 (reference)	
Student	178 (35)	1.16 (0.83–1.63)	.37	1.16 (0.80–1.69)	.43
Unemployed or welfare payment	219 (40)	1.44 (1.06–1.93)	.02	1.29 (0.94–1.78)	.11
Early retirement^c^	167 (50)	2.15 (1.55–2.99)	<.001	1.63 (1.12–2.38)	.01
Retirement	298 (50)	2.18 (1.68–2.83)	<.001	1.88 (0.79–4.47)	.15
Other^d^	467 (34)	1.1 (88.0–1.39)	.40	1.13 (0.85–1.50)	.41
Data missing	0 (0.0)				
**Household income**					
Low tertile	806 (37)	1 (reference)		1 (reference)	
Middle tertile	806 (35)	0.88 (0.71–1.09)	.23	0.89 (0.69–1.14)	.34
High tertile	805 (37)	0.99 (0.80–1.23)	.94	1.04 (0.81–1.35)	.75
Data missing	15 (0.6)				
**Education**					
Lower secondary school	624 (36)	1 (reference)		1 (reference)	
Upper secondary school	826 (37)	1.01 (0.81–1.25)	.96	1.15 (0.91–1.47)	.42
Postsecondary school	363 (45)	1.51 (1.16–1.96)	.002	1.62 (1.22–2.15)	.001
Unknown	619 (33)	0.88 (0.70–1.12)	.30	1.18 (0.90–1.55)	.23
Data missing	0 (0.0)				
**Household crowding**					
≤20 m^2^ per person	2092 (37)	1 (reference)		1 (reference)	
>20 m^2^ per person	237 (40)	0.88 (0.67–1.16)	.37	0.82 (0.55–1.23	.34
Data missing	103 (4.2)				
**Population density**					
0–75 inhabitants/km^2^	802 (30)	1 (reference)		1 (reference)	
76–159 inhabitants/km^2^	1,239 (37)	1.28 (1.05–1.56)	.01	1.35 (1.08–1.68)	.008
160–900^+^ inhabitants/km^2^	379 (47)	1.92 (1.49–2.48)	<.001	2.04 (1.5–2.76)	<.001
Data missing, no. (%)	15 (0.6)				
**Length of residence in Denmark**					
≤5 y	513 (31)	1 (reference)			
>5 y	1904 (38)	1.37 (1.11–1.69)	.003	1.11 (0.85–1.43)	.45
Data missing	15 (0.6)				

Note. MRSA, methicillin-resistant *Staphylococcus aureus*; OR, odds ratio; CI, confidence interval; aOR, adjusted OR.

a Decolonization treatment: mupirocin nasal ointment and body wash using chlorhexidine soap 4% for 5–10 days. Successful decolonization treatment. MRSA-negative follow-up swab samples from nose and throat (same date) at least 6 months after completion of treatment to the end of the 2-year follow-up period.

b Adjusted for age, sex, number of decolonization treatments, MRSA-diagnosed in the household, and throat carriage.

c Voluntary early retirement of individuals partly depending on self-financing and age >60 y.

d Other includes individuals who have no connection to the labor market or with little connection to the labor market. Individuals in the other group do not receive welfare payments or education. Furthermore, children aged <5 years are included in this group.

## Discussion

In this cohort study, we evaluated decolonization treatment for MRSA carriage in 2,536 individuals over a study period of 13 years. The adherence to posttreatment follow-up swab sampling after 1 month was 66%, which decreased to 30% after 6 months. Retirement and living in predominantly urban and intermediate municipalities were associated with significantly higher rates of adherence to posttreatment follow-up swab sampling. The MRSA clearance rate after decolonization treatment was 36% 2 years after initiating treatment. Living in predominantly urban and intermediate municipalities, having retired early, and having a higher education were associated with more successful rates of decolonization treatment.

Individuals who had completed postsecondary school were likelier to achieve successful decolonization treatment. Higher education is associated with characteristics such as health literacy, prestige, and problem-solving skills, which have important health effects.^10^ A substantial proportion of the Danish population perceives difficulties related to understanding health information and engaging with healthcare providers.^30^ MRSA studies indicate that patients actively seek information about decolonization treatment when help-desk access is provided and that information increases knowledge about MRSA.^31,32^ Furthermore, individuals who can retire early might have additional advantages such as time and still being healthy, thereby explaining why this group achieves a higher level of MRSA clearance.

Interestingly, living in predominantly urban municipalities was associated with a higher rate of adherence to posttreatment control sampling and a higher success rate of MRSA clearance. This finding may be due to the benefits of an urban living environment outweighing the possible higher risk of MRSA transmission in crowded communities. Low-quality health care is a frequent problem in rural areas. Numerous problems associated with low-quality healthcare in rural areas emerge as deficiencies in the provision of continued and coordinated care. Problems occur because of shortcomings in interprofessional communication, a general lack of resources, or patients requiring more specialized care.^33^ Some of the predominantly rural municipalities in our study have been struggling with shortages of physicians in hospitals and among general practitioners.

In our study, we obtained a MRSA clearance rate of 36%. A causal effect of decolonization treatment on MRSA clearance has been reported in 2 small studies of selected MRSA-positive patients. A Swedish study found a success rate after 6 months of 61% among patients treated once with topical treatment and systemic antibiotics. Patients receiving topical treatment had a success rate of 13%.^34^ In another study, the success rates were 74% in the treated group and 32% in the untreated group after 3 months.^35^ The low long-term decolonization effect of 36% in our study cohort may be partly explained by the low number (11.6%) of individuals receiving systemic antibiotics together with the topical treatment. Long-term decolonization effects, the impact of repeated courses of treatment, possible side effects of decolonization treatments evaluated in randomized controlled trials, and organizational matters remain understudied.^36^ More successful decolonization treatment has been reported when MRSA management is handled by a specialist team or in an outpatient setting with close cooperation with the inpatient sector, but none of these studies had control groups, and the aim of the studies was not to compare organizational differences.^37,38^

One strength of this study is that it comprises a large community cohort based on individual data and an observational period of 2 years for each person included. However, this study has some limitations that should be considered when interpreting our findings. First, we included only registry data on the prescription of mupirocin nasal ointment to estimate the completed decolonization treatment. Clinical data from general practice were not routinely transferred to national registries in Denmark. This limited our ability to determine other factors that may have contributed to the observed difference such as validation of the data collection method for initiated and completed decolonization treatment. Furthermore, we could not measure sociocultural differences or stage-of-life characteristics. Second, some of the socioeconomic measures considered were highly correlated with each other. For example, a high education level could be correlated with other socioeconomic factors, such as a high income. This raises concerns about collinearity. However, previous evidence has shown that the correlation between different socioeconomic factors was not strong enough to be grouped as 1 measurement.^10^ Third, ∼50% of MRSA-diagnosed individuals were excluded due to no treatment. The sensitivity analysis revealed that decolonization treatment was less likely to be initiated if the individuals were males, were employed, lived in predominantly rural municipalities, and had recently immigrated to Denmark. The RSD is a high-density farming area and persons with day-to-day contact with live pigs do not initiate decolonization treatment, which may explain some of the high proportions of untreated individuals. However, limited access to healthcare in rural areas is frequently reported.^33^ Furthermore, immigrants with a risk of severe disease from MRSA may be less likely to initiate treatment. In a Danish study, mortality rates from infectious diseases were higher among refugees and immigrants compared to native Danes. The study also stated that a lack of information on consulting general practitioners and language barriers may impair access to healthcare services.^39^ Furthermore, individuals who have recently immigrated are often placed in rural municipalities, which challenges mobilization.^40^ This raises a concern for inequity in MRSA morbidity and mortality for individuals who have immigrated to Denmark within the last 5 years. Finally, a few associations in our study were relatively weak (adjusted OR < 1.5), though it is difficult to assess the clinical significance because research on the number needed to treat to reduce infections or to prevent transmission of MRSA in the community is lacking in the literature.^36^

To generalize the findings on rural areas it should be noted that most travel distances are short in Denmark.^12^ Rural districts within the municipalities often have a maximum of 30 minutes of driving by car to the nearest general practitioner or hospital.

Our findings have some implications for future research. The surprising benefits of living in predominantly urban municipalities require further study. Furthermore, it is unclear whether the low rate of follow-up sampling and successful decolonization treatment in our study was due to the data collection method, the present organization of MRSA care in primary healthcare, or ineffective decolonization treatment regimens.

In conclusion, we found low rates of adherence to MRSA follow-up sampling and low rates of successful decolonization treatment. Lower decolonization rates and lower adherence to follow-up tests were not linked to overcrowding, low income, unemployment, or recent immigration. Further, disparity in the effect of decolonization treatment and adherence to MRSA follow-up sampling among MRSA-positive individuals appear largely explained by the level of education, area of residence in predominantly urban and intermediate municipalities, and employment status.

## Supplementary material

For supplementary material accompanying this paper visit https://doi.org/10.1017/ice.2023.32. click here to view supplementary material
