# Supplementary material for: The association between socioeconomic factors and the success of decolonization treatment among individuals diagnosed with methicillin-resistant Staphylococcus aureus: A cohort study from 2007 to 2020
Source: Infect Control Hosp Epidemiol. 2023 Apr 5;44(10):1620–8. doi: 10.1017/ice.2023.32 (PMC10587379; doi:10.1017/ice.2023.32)
Supplement: Supplementary file 1 [file S0899823X23000326sup.zip › S0899823X23000326sup002.pdf]

Supplementary File 2. Baseline[a] demographic and socioeconomic characteristics of treated[b] or non-treated methicillin-resistant *Staphylococcus aureus* positive individuals

	Number (%)		
N=(4,451)	Treated (n=2,550)	Non-treated (n=1,901)	P-value[c]
Age group			0,000
0-34 years	1,330 (52)	1,098 (58)	
35-49 years	437 (17)	392 (21)	
50-64 years	427 (17)	217 (11)	
65-79 years	269 (11)	111 (6)	
80^{+} years	74 (3)	42 (2)	
Sex			0,000
Male	1,218 (48)	1,170 (62)	
Female	1,319 (52)	690 (36)	
Missing	13	41	
Employment status			0,000
Employed	1,183 (46)	1,080 (57)	
Student	177 (7)	97 (5)	
Unemployed or welfare payment	203 (8)	109 (6)	
Early retirement[d]	176 (7)	63 (3)	
Retirement	313 (12)	134 (7)	
Other[d]	497 (19)	411 (22)	
Data missing	1 (0,04)	7 (0,4)	
Household income			0,000
Low tertile	835 (33)	631 (33)	
Middle tertile	856 (34)	610 (32)	
High tertile	846 (33)	619 (33)	
Data missing	13 (0,5)	41 (2)	
Education			0,000
Lower secondary school	670 (26)	413 (22)	
Upper secondary school	862 (34)	650 (34)	
Post-secondary school	373 (15)	234 (12)	
Unknown	645 (25)	604 (32)	
Household crowding			0,001
≤ 20 m^2 per person	232 (9)	162 (9)	
> 20 m^2 per person	2179 (85)	1,584 (83)	
Data missing	139 (5)	155 (8)	

Population density			0,000
0-75 inhabitants per square kilometer	849 (33)	693 (36)	
76-159 inhabitants per square kilometer	1,287 (50)	913 (48)	
160-660 inhabitants per square kilometer	401 (16)	254 (13)	
Data missing	13 (0,59	41 (2)	
Length of residence in Denmark			0,000
≤ 5 years	553 (22)	583 (31)	
> 5 years	1984 (78)	1,277 (67)	
Data missing	13 (0,5)	41 (2)	

a: First positive MRSA test registered for an individual
b: Decolonization treatment: Mupirocin nasal ointment 2% and body wash using chlorhexidine soap 4 % for five or ten days (throat carriage)
c: Chi-squared test
d: Voluntary early retirement of individuals depending on partly self-financing and an age above 60.
e: Other includes individuals who have no connection to the labor market or with little connection to the labor market. Individuals in the other group do not receive welfare payments or education. Furthermore, children under the age of five years are in this group.
