# Supplementary figures and images for: The association between socioeconomic factors and the success of decolonization treatment among individuals diagnosed with methicillin-resistant Staphylococcus aureus: A cohort study from 2007 to 2020
Source: Infect Control Hosp Epidemiol. 2023 Apr 5;44(10):1620–8. doi: 10.1017/ice.2023.32 (PMC10587379; doi:10.1017/ice.2023.32)

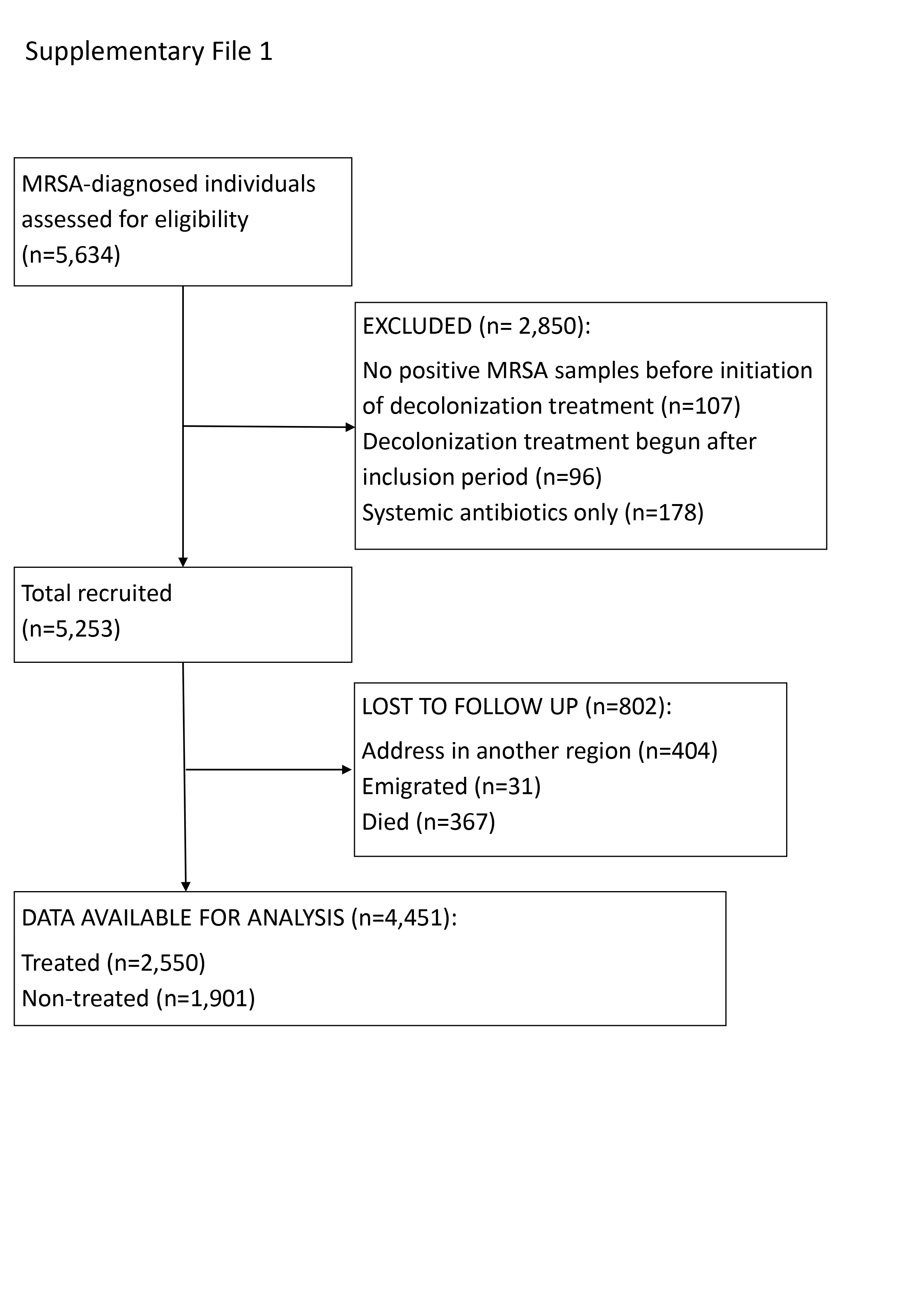

Supplement: Supplementary file 1 [file S0899823X23000326sup.zip › S0899823X23000326sup001.tiff]
